# The effect of vacation on reading fluency development

**DOI:** 10.1590/2317-1782/e20250003en

**Published:** 2025-12-01

**Authors:** Isa Mourão Carvalho, Luiza Vitória da Cunha Ferreira, Rebecca Chrispim Silva, Dayanne Gabrielle da Cruz Oliveira, Letícia Correa Celeste, Luciana Mendonça Alves

**Affiliations:** 1 Programa de Pós-graduação em Ciências Fonoaudiológicas, Faculdade de Medicina, Universidade Federal de Minas Gerais – UFMG - Belo Horizonte (MG), Brasil.; 2 Faculdade de Medicina, Universidade Federal de Minas Gerais – UFMG - Belo Horizonte (MG), Brasil.; 3 Curso de Fonoaudiologia, Faculdade de Ceilândia, Universidade de Brasília – UnB - Brasília (DF), Brasil.; 4 Departamento de Fonoaudiologia, Faculdade de Medicina, Universidade Federal de Minas Gerais – UFMG - Belo Horizonte (MG), Brasil.

**Keywords:** Reading, Learning, Evaluation Study, Holidays, Students

## Abstract

**Purpose:**

To evaluate the effect of vacations on the reading fluency of elementary school students.

**Methods:**

This analytical, longitudinal, observational study included 98 students from 2nd to 5th grades, who read texts appropriate for their school year before and after vacations between 2022 and 2024. The performance of the same students in both periods and the progression of reading speed between grades throughout the year were compared. The paired t-test was used to assess reading performance at different time points, the t-test to analyze progression, and Cohen's d test to calculate the effect size.

**Results:**

The findings show an improvement in reading speed measures between the beginning and end of the school year. However, they also point to a statistically significant difference between before and after extended vacations, indicating a decline in post-vacation performance – except for students going from 2nd to 3rd grade, whose reading practice remained constant. The findings reinforce the importance of pedagogical strategies to encourage continued reading during vacation.

**Conclusion:**

This study highlighted the negative impact of the vacation effect on reading fluency performance among 3rd-, 4th-, and 5th-grade students, whose reading fluency declined after the extended school vacation.

## INTRODUCTION

The phenomenon known as summer learning loss or summer reading loss is characterized by a decline in academic performance among students due to extended vacations. This phenomenon has been widely studied worldwide due to its negative impact on the learning process in various areas of knowledge^(1-6)^.

The Brazilian school year ends and begins in December and February, respectively. Between these months, students are on summer vacation (the longest recess in Brazil) for rest and leisure, when they tend to decrease their reading practice, resulting in a poorer reading fluency performance^(3,7)^.

Individual development is largely influenced by reading, which impacts their social, personal, and academic performance. Learning to read requires skills such as decoding, sound and syllable manipulation, word recognition, and language comprehension^(8,9)^. Reading fluency improves as these skills evolve and exposure to texts increases^(7)^.

Reading fluency is characterized by continuous and effortless reading^(10,11)^ and is evidenced as a predictor of academic success^(12-15)^. It is assessed based on three dimensions: accuracy, speed, and expressiveness. Despite being subject to individual identification and assessment, they are integrated into the continuum of reading skill^(10)^, commonly measured by words read per minute (WPM), which quantifies reading speed, and words correct per minute (WCPM), which quantifies reading accuracy^(16)^.

Accuracy refers to the precision in decoding what is read. When reading is done accurately, the reader requires less effort to decode the message, favoring the development of automaticity. The latter, directly associated with reading speed, is an important prerequisite for greater expressiveness, allowing the reader to convey attitudes and intentions, highlighting the meanings learned from the text^(11,12,17)^.

The combination of these three factors (accuracy, speed, and expressiveness) has a significant impact on reading comprehension^(11,12,17)^. Readers with well-developed skills expend less effort decoding words, allowing them greater cognitive availability to focus on the semantic content of the text^(9,16)^.

Readers need to be exposed to reading practices in their daily lives to improve fluent reading skills^(7,12)^. Studies show that reduced cognitive stimulation during the holidays can impact the development of reading fluency and other academic skills, such as mathematics^(1)^, requiring teachers to spend class time reviewing previously developed content before moving on to other topics^(5)^.

The literature also indicates that this loss in academic skills performance can also vary according to factors such as socioeconomic status, educational level, and previous reading difficulties^(3,18,19)^. Menard and Wilson’s study^(3)^ showed that students who already had reading difficulties before the summer vacation had a more pronounced regression in their reading skills during this period than students who did not face such difficulties.

Given the importance of reading fluency in students' lives, measuring and monitoring this skill should be systematic, especially during elementary school. This is justified by the fact that the automaticity of reading begins in the third grade, when students reach an intermediate reading level^(15)^.

There is a lack of Brazilian research that portrays the impact of the vacation effect on schoolchildren’s reading fluency. This highlights the importance of investigating the impact of this phenomenon on the reading fluency performance of Brazilian students to expand knowledge of reading practice in Brazil and propose actions aimed at minimizing this effect on our students’ learning.

Thus, this study aimed to evaluate the effect of vacations on the reading fluency of elementary school students.

## METHODS

### Participants

This is an analytical, longitudinal, observational study with a school and sample selected for convenience, approved by the Research Ethics Committee of the researchers' affiliated institution under approval number 5,735,604.

The research took place at a private school in Belo Horizonte, Minas Gerais, Brazil, in December 2022 (before vacation), March 2023 (after vacation), December 2023 (before vacation), and March 2024 (after vacation). Approximately 72% of this school’s students have both parents with a bachelor’s degree, and 91% have at least one parent with a bachelor’s degree.

**Figure 1:**
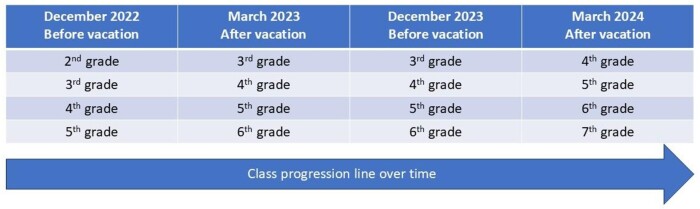
Data collection progression line in four time points with two vacations

The study included elementary school students from the 2^nd^ to the 5^th^ grade. They were in the 2^nd^ to 5^th^ grade in the collection carried out in December 2022; in the 3^rd^ to 6^th^ grade in 2023; and in the 4^th^ to 7^th^ grade in 2024. This progression is shown in Figure 1.

The eligibility criteria for this study sample were enrollment in the same participating school during the research collection period and the signature of an informed consent form by parents and of an informed assent form by participants. The exclusion criterion was non-participation in any assessment (before or after vacation). Student selection was based on their and their parents’ willingness to participate in the assessment.

### Sample characterization

This study included 98 elementary school students from the 2^nd^ to 5^th^ grade who underwent reading fluency assessments in December 2022, March and December 2023, and March 2024. Of these 98 students, 31 were in the 2^nd^ grade, 23 in the 3^rd^ grade, 15 in the 4^th^ grade, and 24 in the 5^th^ grade.

### Procedures

The readings were recorded in person, individually, in the school’s library and/or in rooms away from noise sources. A laptop connected to a unidirectional microphone and Praat software were used to record and synthesize the audio recordings of the students' oral readings (aloud). Before starting, participants were instructed to read the text aloud, beginning with the title. Any questions about the text or how to read it were clarified. The students then performed the reading.

The oral reading assessment used texts from the “*Avaliação da compreensão leitora de textos expositivos*” (Assessment of Reading Comprehension of Expository Texts)^(20)^, consisting of two standardized texts appropriate for each school year. The texts presented to the students were selected according to each group's grade; the text with the fewest words was chosen for the beginning of the year, and the slightly longer text was used for the end-of-year assessment. Thus, in 2022, 2^nd^-graders read a text appropriate for their level. The following year (2023), in 3^rd^ grade, the same students read the two texts corresponding to this level. In 2024, in 4^th^ grade, the class read one of the texts assigned for that grade. To avoid the influence of prior contact with the text, a new text was administered in each assessment.

**Figure 2:**
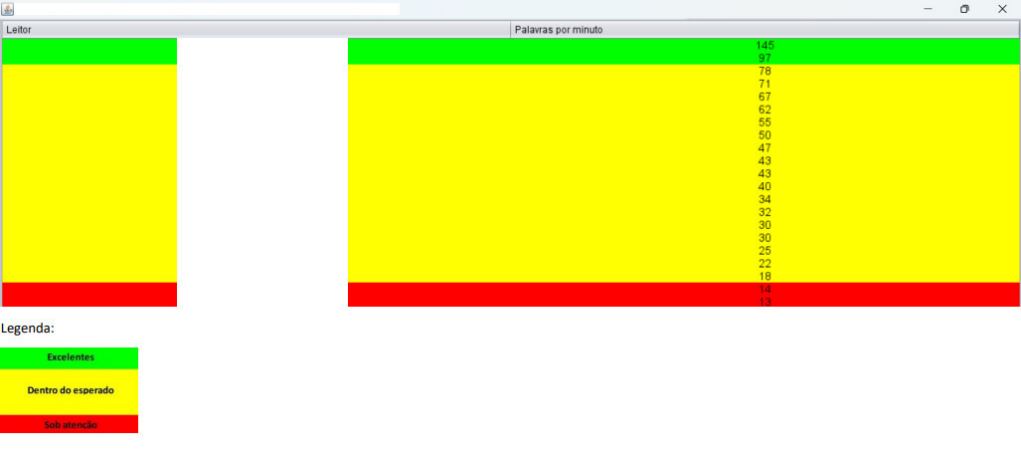
Graphical example of performance by class, with reading speed measurements, generated by Lepic software. Student names are protected under the white bar

WPM (reading speed) was analyzed in the reading recordings, using Lepic software^(21)^. This tool generates a graph representing the classes' performance, accompanied by a standard deviation measure that classifies readers according to the classes’ average performance as excellent (above the standard deviation), within the expected range (within the standard deviation), and under observation (below the standard deviation). Figure 2 shows an example of the graph generated by the software. The data displayed represent the reading speed results of a class at a specific assessment time, demonstrating the program's functioning. The results in WPM were stored in an Excel spreadsheet.

Caption: *Leitor* = Reader; *Palavras por minuto* = Words per minute; *Legenda* = Caption; *Excelentes* = Excellent; *Dentro do esperado* = Within the expected range; *Sob atenção* = Under observation

A descriptive analysis of the data was performed using measures of central tendency (mean) and variability (standard deviation) for the continuous variable (reading speed – WPM). The normality of the distribution of continuous variables was assessed using the Shapiro-Wilk test, which indicated a normal distribution for the study variable.

The analysis approached the reading fluency performance of elementary school students from the 2^nd^ to 5^th^ grades. The same students’ performances were compared between December 2022 and March 2023, March 2023 and December 2023, and December 2023 and March 2024, as well as the progression of reading speed between school grades. In the collection carried out in March 2023 and 2024, respectively, the students were in the 3^rd^ to 6^th^ grades and from the 4^th^ to 7^th^ grades. The analysis used the paired t-test to assess reading performance at different time points and the t-test to analyze progression, considering a 0.05 significance level. School grades were grouped to calculate the effect size, and the difference between periods was calculated in terms of standard deviation using Cohen's d test, interpreted as follows^(22)^:

- 0.00 - 0.19: Very small.
- 0.2 - 0.49: Small.
- 0.5 - 0.79: Medium.
- 0.80 or greater: Large.

## RESULTS

### Reading performance

Tables 1, 2, and 3 present the students’ reading speed performance during the evaluated periods. Tables 1 and 2 show statistically significant differences between their performances before and after vacation, indicating a decline in performance between December 2022 (before vacation) and March 2023 (after vacation), and between December 2023 (before vacation) and March 2024 (after vacation).

**Table 1:** Student performance in words per minute in December 2022 and March 2023

School grade	N	Assessment time	Mean	Standard deviation	WPM		
					Statistics	Degrees of freedom	p-value*
**2^nd^ grade**	31	**Dec 22**	88.9	25.5	-2.11	30.0	0.043
		**Mar 23**	94.2	26.8			
**3^rd^ grade**	23	**Dec 22**	112.4	30.5	7.14	22.0	<0.001
		**Mar 23**	88.4	23.1			
**4^th^ grade**	15	**Dec 22**	136.3	17.5	6.14	14.0	<0.001
		**Mar 23**	112.1	19.0			
**5^th^ grade**	29	**Dec 22**	140.3	22.3	10.39	28.0	<0.001
		**Mar 23**	113.5	20.1			

Caption: N = number of students

* paired t-test

**Table 2:** Student performance in words per minute in December 2023 and March 2024

School grade	N	Assessment time	Mean	Standard deviation	WPM		
					Statistics	Degrees of freedom	p-value*
**4^th^ grade**	31	**Dec 23**	108.6	20.6	12.22	30.0	<0.001
		**Mar 24**	88.8	21.6			
**5^th^ grade**	23	**Dec 23**	116.2	26.1	4.81	22.0	<0.001
		**Mar 24**	103.6	22.5			
**6^th^ grade**	15	**Dec 23**	140.5	19.6	6.63	14.0	<0.001
		**Mar 24**	124.0	18.0			
**7^th^ grade**	29	**Dec 23**	134.5	20.6	3.46	28.0	0.002
		**Mar 24**	128.2	19.1			

Caption: N = number of students

* paired t-test

**Table 3:** Student performance in words per minute in March and December 2023

School grade	N	Assessment time	Mean	Standard deviation	WPM		
					Statistics	Degrees of freedom	p-value*
**3^rd^ grade**	31	**Mar 23**	94.2	26.8	-5.60	30.0	<0.001
		**Dec 23**	108.6	20.6			
**4^th^ grade**	23	**Mar 23**	88.4	23.1	-10.64	22.0	<0.001
		**Dec 23**	116.2	26.1			
**5^th^ grade**	15	**Mar 23**	112.1	19.0	-10.13	14.0	<0.001
		**Dec 23**	140.5	19.6			
**6^th^ grade**	29	**Mar 23**	113.5	20.1	-9.21	28.0	<0.001
		**Dec 23**	134.5	20.6			

Caption: N = number of students

* paired t-test

In Table 3, the analysis compares the results from March and December 2023 and shows an improvement in student performance between the beginning and the end of the school year.

**Figure 3:**
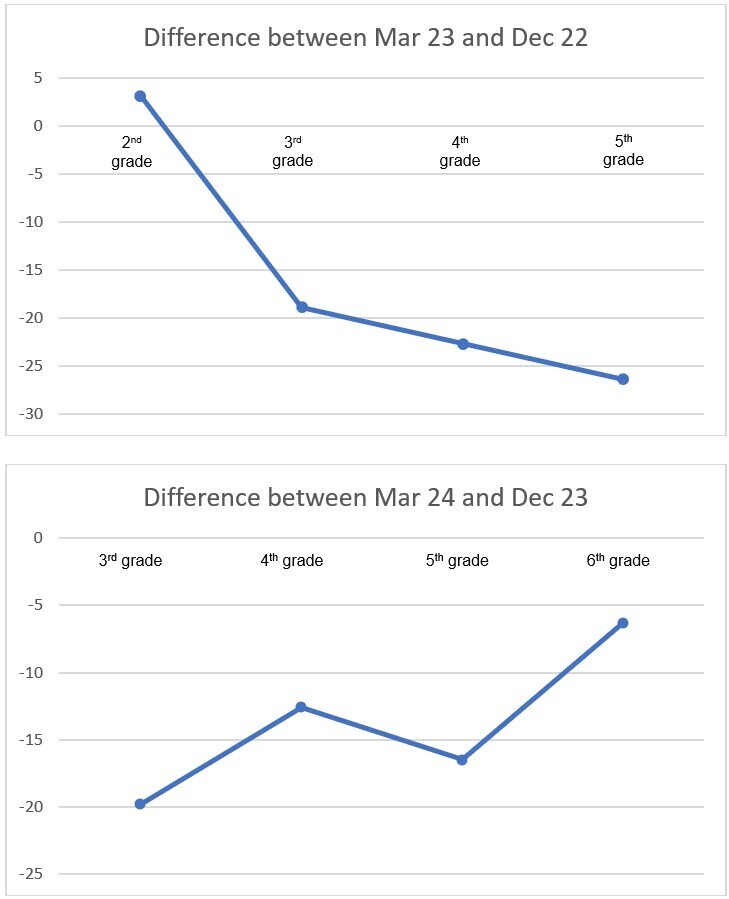
Difference between reading fluency performance between after vacation (March 2023 and March 2024) and before vacation (December 2022 and December 2023) per school grade

The summer learning loss is seen in Figure 3, in which the values ​​of March 2023 (after vacation) were subtracted from those of December 2022 (before vacation) (2^nd^ to 5^th^ grades), and those of March 2024 (after vacation) were subtracted from those of December 2023 (before vacation) (3^rd^ to 6^th^ grades).

### Progression of reading fluency

**Figure 4:**
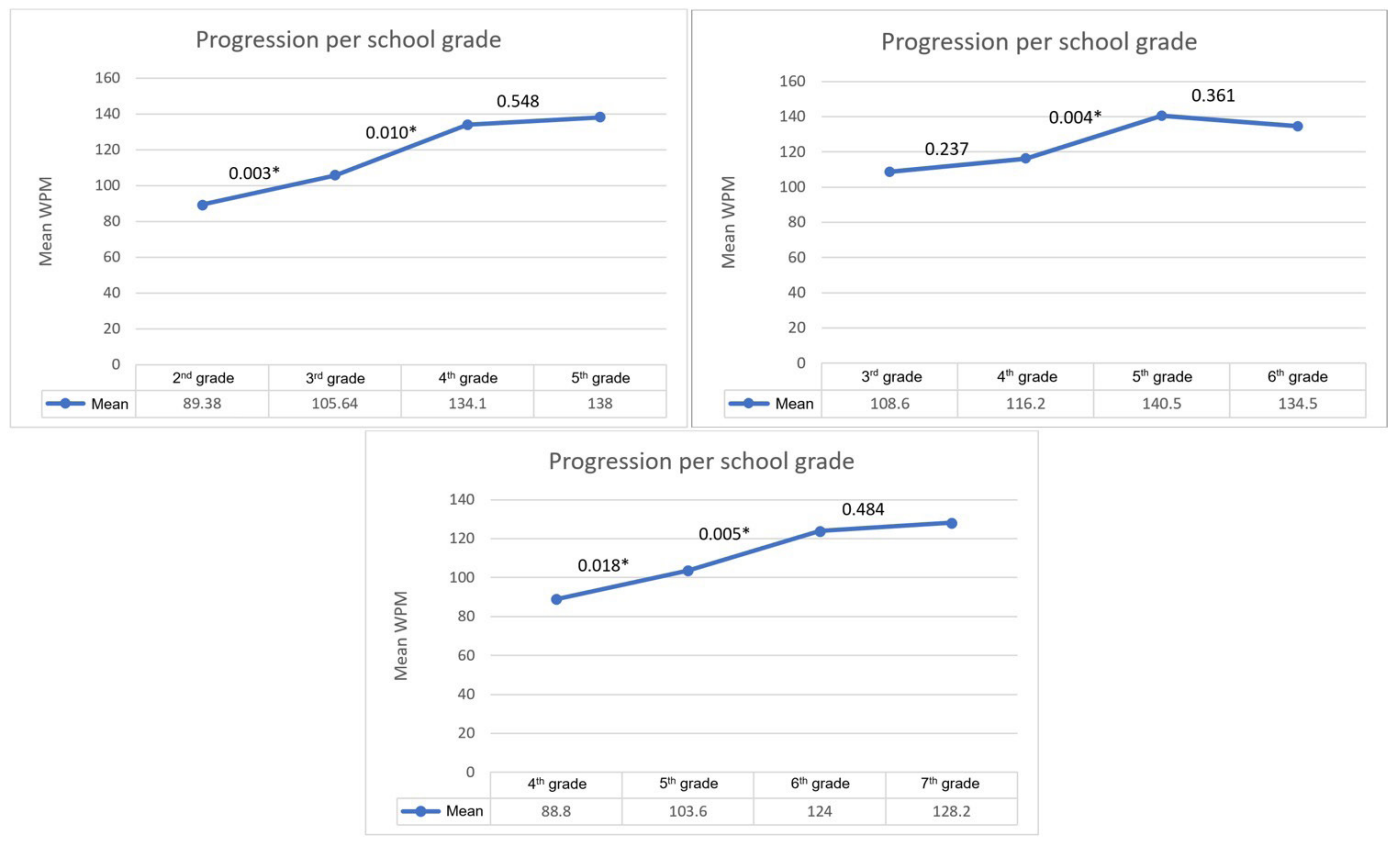
Comparison of reading speed between school grades in December 2022, December 2023, and March 2024, respectively

Figure 4 shows the progression of reading skills according to the school grade in 2022, 2023, and 2024. In 2022, the results are statistically significantly different in the comparisons between the 2^nd^ and 3^rd^ grades and between the 3^rd^ and 4^th^ grades. Regarding the 2023 results, in which the previous year's students were between the 3^rd^ and 6^th^ grades, a statistically significant difference is only seen in the comparison between the 4^th^ and 5^th^ grades. In 2024, the results indicate a statistically significant difference between the 4^th^ and 5^th^ grades and between the 5^th^ and 6^th^ grades.

Caption: *p-values with statistical significance (Student’s t-test); p-value < 0.05

### Effect size

As shown in the table below, the effect size in the differences between groups had greater weight in the comparison between March and December 2023.

## DISCUSSION

This study aimed to evaluate the effect of vacations^(5,6,19)^ on the process of developing reading fluency among elementary school students at a private school in Belo Horizonte, Minas Gerais, Brazil.

Although vacations can provide opportunities to stimulate and strengthen reading fluency development, some students lack this stimulation. This downtime can lead to a temporary decrease in reading practice, which can negatively impact reading fluency^(3,4)^.

This study found a decrease in reading speed between before and after vacation. Tables 1 and 2 show that the reading speed of the 2^nd^ grade remained stable. However, the 3^rd^, 4^th^, and 5^th^ grades worsened significantly (p < 0.05), which is assumed to be associated with the vacation effect^(5)^. This result was also observed by Alves et al.^(7)^ and Menard and Wilson^(3)^, whose studies describe the reduction in reading speed after the vacation, associated with both the lack of practice and less exposure to texts.

From another perspective, goal 5 of the National Literacy Plan (PNE)^(23)^ requires all children to be literate by the end of the 3^rd^ grade at the latest. Therefore, the 2^nd^ grade, having recently learned to read, may have received greater encouragement from their parents to practice reading during vacation. Thus, the fact that this class’s performance did not decline after vacation can be explained by the greater encouragement and exposure to reading during this period. Furthermore, these students learned to read in kindergarten or the 1^st^ grade during the COVID-19 pandemic, through remote classes, which resulted in greater monitoring and attention from families and schools to maintain their reading level. Thus, maintaining reading fluency in this population is justified.

Figure 3 shows the difference between the averages for December 2022 and March 2023 and between December 2023 and March 2024. Vacation initially had a smaller impact in the second year, but it became more pronounced in the subsequent year. This phenomenon may indicate a variation in the continuity or intensity of educational activities between vacations. In other words, if the student stopped receiving any family support or encouragement between December 2023 and March 2024, this may have helped to mitigate the negative effects associated with vacation, worsening their reading fluency performance^(1,7)^.

Despite the decline in students' reading performance during vacation, Table 3 shows that these same students improved their skills throughout the year. Comparing the results between March and December 2023, all school grades achieved superior results, with a statistically significant difference in the second assessment, which is expected according to the literature^(11,24)^.

Tables 1, 2, and 3 show a higher WPM average as school grades progressed – i.e., their reading speed improved^(11)^, as reinforced by Figure 4. In the first graph, with the 2022 results, there is a statistically significant difference between the 2^nd^ and 3^rd^ grades (p = 0.003) and between the 3^rd^ and 4^th^ grades (p = 0.010). In 2023, there is a statistically significant difference between the 4^th^ and 5^th^ grades (p = 0.004). In 2024, there are results with a statistical difference between the 4^th^ and 5^th^ grades (p = 0.018) and between the 5^th^ and 6^th^ grades (p = 0.005). Finally, the last few years had no results with a statistical difference in any of the analyses.

Furthermore, Table 4 shows the effect size results, indicating that the effect was greater in the comparison between March and December 2023 (d = 0.88), which suggests a substantial difference in reading fluency throughout the school year. This finding may reflect the impact of the school grade on the consolidation of reading skills, with significant progress between the beginning and the end of the year.

**Table 4:** Values ​​of the effect size results through Cohen's d test

Assessment time	WPM – Dec 2022	WPM – Mar 2023	WPM – Dec 2023	WPM – Mar 2024
**WPM – Dec 2022**	-	0.52	0.2	0.2
**WPM – Mar 2023**	0.52	-	0.88	0.3
**WPM – Dec 2023**	0.2	0.88	-	0.3
**WPM – Mar 2024**	0.2	0.3	0.3	-

On the other hand, the lower values ​​in the comparisons involving the months of March (ranging from 0.2 to 0.3) indicate a reduced effect or even a possible stagnation in reading performance after the summer vacations. This pattern may be related to the well-known decline in learning during these vacations (summer learning loss), a phenomenon in which skills acquired during the school year tend to regress in the absence of regular practice. These findings corroborate the results mentioned earlier in this study and reinforce the importance of pedagogical strategies that encourage continued reading during vacation to mitigate losses and promote a more efficient resumption of skills at the beginning of the school year.

The discrepancy between school grades highlights the need for further research to better understand the factors that contribute to the stability or decline of reading skills during vacations at different stages of development. Understanding these factors can help educators develop more effective strategies to minimize the impact of vacations on student learning.

The decline in reading skills during vacation is also influenced by socioeconomic context. Studies^(18,19)^ show that the vacation effect is significantly greater in children from low-income backgrounds, which further widens the achievement gap when returning to school^(4)^. Children from families with limited financial resources may have little access to reading materials, such as books and magazines. Furthermore, these families may have less time and resources to devote to educational activities during vacation. As a result, inequalities in access to and participation in reading activities during vacation may widen learning disparities among students from different socioeconomic backgrounds.

This study did not consider the relationship between reading fluency and socioeconomic status, as it was conducted in only one private school. This is, therefore, a limitation. However, this restriction also allows for a more specific analysis of the vacation effect on reading fluency, as the reduced number of variables facilitates observation of this phenomenon. Furthermore, reading habits among the students in the sample were not investigated, which constitutes another limitation of the study.

Comparisons between classes in different grades, such as 2^nd^ and 3^rd^ grade, may not be completely representative, as a difference of just 3 months may not be enough for 2^nd^-grade students to reach the reading proficiency expected for 3^rd^ grade. This may have influenced the measurements and, consequently, the results.

We suggest conducting more robust research, with more representative samples from different types of schools and other regions of the country, to generalize the results of the impact of vacations in Brazil. Similarly, future research should investigate the reading habits of participants and their families.

The discussion of the difference in reading speed stability across grades highlights the complexity of the vacation effect phenomenon and underscores the importance of differentiated approaches to supporting the continued development of students' reading skills at all ages and grades.

The results lead us to reflect on the importance of targeted educational interventions to remedy reading declines during vacation. Moreover, family guidance regarding reading habits is crucial. Strategies such as summer reading programs, access to online educational resources, and individualized support can be crucial for maintaining and strengthening students' reading skills during formal education recesses, provided they are well-planned, have good adherence, and provide sufficient time for learning^(2,19,25-27)^. Thus, disseminating the results of this research can inspire actions to develop this important practice (reading) by encouraging it also during school vacation in a pleasurable way.

## CONCLUSION

This study highlighted the negative impact of the vacation effect on the reading fluency performance of elementary school students, whose skills declined after extended school vacations.

The results from 2^nd^ grade reinforce this finding, as the class initially maintained stable reading fluency scores immediately after learning to read. However, their performance declined in subsequent years, which may be attributed to reduced stimulation and less attention given to these students during vacation.
