# Supplementary material for: The effect of vacation on reading fluency development
Source: Codas. 2025 Dec 1;37(6):e20250003. doi: 10.1590/2317-1782/e20250003en (PMC12704612; doi:10.1590/2317-1782/e20250003en)
Supplement: Translation

# Translation: Efeito das férias sobre o desenvolvimento da fluência leitora

**DOI:** 10.1590/2317-1782/e20250003pt

**Authors:** Isa Mourão Carvalho, Luiza Vitória da Cunha Ferreira, Rebecca Chrispim Silva, Dayanne Gabrielle da Cruz Oliveira, Letícia Correa Celeste, Luciana Mendonça Alves

**Affiliations:** 1 Programa de Pós-graduação em Ciências Fonoaudiológicas, Faculdade de Medicina, Universidade Federal de Minas Gerais – UFMG - Belo Horizonte (MG), Brasil.; 2 Faculdade de Medicina, Universidade Federal de Minas Gerais – UFMG - Belo Horizonte (MG), Brasil.; 3 Curso de Fonoaudiologia, Faculdade de Ceilândia, Universidade de Brasília – UnB - Brasília (DF), Brasil.; 4 Departamento de Fonoaudiologia, Faculdade de Medicina, Universidade Federal de Minas Gerais – UFMG - Belo Horizonte (MG), Brasil.

**Keywords:** Leitura, Aprendizagem, Estudo de Avaliação, Férias e Feriados, Estudantes

## Abstract

**Objetivo:**

avaliar o efeito das férias na fluência leitora de escolares do Ensino Fundamental I.

**Método:**

estudo observacional longitudinal analítico. 98 escolares do 2° ao 5° ano do Ensino Fundamental realizaram a leitura de textos adequados à escolaridade antes e após as férias entre 2022 e 2024. Comparou-se o desempenho dos mesmos alunos nos dois períodos e a progressão da velocidade de leitura entre as séries durante o ano. Utilizou-se o teste T pareado para a avaliação do desempenho de leitura nos diferentes momentos, o teste T para a análise da progressão, e para calcular o tamanho de efeito, o teste d de Cohen.

**Resultados:**

Os achados evidenciam uma melhora nas medidas de velocidade de leitura entre o início e o fim do ano escolar. No entanto, também apontam diferença com relevância estatística ao comparar pré e pós férias prolongadas, indicando declínio do desempenho pós-férias, exceto para os alunos na transição do 2º para o 3º ano, em que as práticas de treino de leitura se mantiveram constantes. Os achados reforçam a importância de estratégias pedagógicas que incentivem a manutenção da leitura durante as férias.

**Conclusão:**

O presente estudo evidenciou o impacto negativo do Efeito das Férias no desempenho da fluência leitora nos escolares do 3°, 4° e 5° anos Ensino Fundamental I, que apresentaram um declínio na habilidade após o período das férias escolares prolongadas.

## INTRODUÇÃO

O fenômeno conhecido como *Summer Learning Loss* ou *Summer Reading Loss* (Declínio da leitura pós-férias) é caracterizado pela piora do desempenho das habilidades acadêmicas de escolares em razão das férias prolongadas. Esse fenômeno tem sido amplamente estudado em âmbito internacional devido ao seu impacto negativo no processo de aprendizagem de diversas áreas de conhecimento^(1-6)^.

No Brasil, o encerramento e o início do ano letivo acontecem, respectivamente, em dezembro e em fevereiro e, nesse intervalo, os escolares se encontram em período de férias de verão – o período de recesso mais extenso no Brasil –, um momento de descanso e lazer para as crianças. Durante esse período, há uma tendência à diminuição das práticas de leitura, ocasionando uma redução no desempenho da fluência leitora^(3,7)^.

O desenvolvimento do indivíduo é amplamente influenciado pela leitura, que impacta seu desempenho social, pessoal e acadêmico. O processo de aprendizagem da leitura exige competências como a decodificação, a manipulação de sons e sílabas, além das habilidades de reconhecimento de palavras e de compreensão da linguagem^(8,9)^. A fluência leitora é aperfeiçoada com a evolução dessas habilidades e o aumento da exposição a textos^(7)^.

A fluência leitora é caracterizada pela leitura contínua e sem esforço^(10,11)^ e é evidenciada como uma competência preditora do sucesso acadêmico^(12-15)^. Ela é avaliada a partir de três dimensões: acurácia, velocidade e expressividade, que, embora passíveis de identificação e avaliação individual, estão integrados no *continuum* da habilidade leitora^(10)^. Essa habilidade é comumente mensurada pelos parâmetros: Palavras Lidas Por Minuto (PPM), que quantifica a velocidade de leitura, e Palavras Lidas Corretamente Por Minuto (PCPM), que quantifica a acurácia de leitura^(16)^.

A acurácia refere-se à precisão na decodificação do que é lido. Quando a leitura é realizada com precisão, o leitor demanda menos esforço para decodificar a mensagem, favorecendo o desenvolvimento da automaticidade. Essa automaticidade, diretamente associada à velocidade da leitura, é um importante pré-requisito para uma maior expressividade, permitindo que o leitor transmita atitudes e intenções, evidenciando os significados apreendidos do texto^(11,12,17)^.

A combinação desses três fatores – acurácia, velocidade e expressividade – exerce um impacto significativo na compreensão leitora^(11,12,17)^. Leitores com essas habilidades bem desenvolvidas despendem menos esforço na decodificação das palavras, o que lhes permite maior disponibilidade cognitiva para se concentrar no conteúdo semântico do texto^(9,16)^.

Para aprimorar a habilidade de ler fluentemente, o leitor precisa ser exposto às práticas de leitura no cotidiano^(7,12)^. Nesse sentido, estudos evidenciam que a redução da estimulação cognitiva durante o período das férias pode impactar no desenvolvimento da fluência leitora e de outras habilidades acadêmicas, como a matemática^(1)^, exigindo que os professores despendam tempo de aula revisando o conteúdo previamente desenvolvido antes de avançar em outros temas^(5)^.

Além disso, a literatura indica ainda que essa perda no desempenho das habilidades acadêmicas também pode variar de acordo com fatores como o índice socioeconômico, o nível de ensino e a existência prévia de dificuldades na leitura^(3,18,19)^. Menard e Wilson^(3)^ evidenciaram em seu estudo que escolares que já apresentavam dificuldades de leitura antes das férias de verão apresentaram uma regressão mais acentuada em suas habilidades de leitura durante esse período, em comparação a estudantes que não enfrentavam tais dificuldades.

Dada a relevância da fluência leitora na vida dos escolares, a mensuração e monitoramento dessa competência deve ocorrer de forma sistemática, especialmente durante os anos iniciais do Ensino Fundamental. Isso se justifica pelo fato de que a automatização da leitura tem início no terceiro ano escolar, período em que o estudante alcança a leitura intermediária^(15)^.

Há uma escassez de pesquisas que retratam o impacto do Efeito das Férias na fluência leitora de escolares em âmbito nacional, o que evidencia a importância de investigar o impacto desse fenômeno no desempenho da fluência leitora dos alunos brasileiros, a fim de ampliar o conhecimento da prática de leitura no Brasil e propor ações voltadas para minimizar este efeito sobre a aprendizagem dos nossos escolares.

Portanto, o objetivo deste trabalho é avaliar o efeito das férias na fluência leitora de escolares do Ensino Fundamental I.

## MÉTODO

### Participantes

Trata-se de estudo observacional longitudinal analítico, com escola e amostra selecionadas por conveniência, aprovado pelo COEP da Instituição de vínculo dos pesquisadores sob parecer número 5.735.604.

A pesquisa ocorreu em uma escola particular em Belo Horizonte, Minas Gerais, durante os meses de dezembro de 2022 (pré-férias), março de 2023 (pós-férias), dezembro de 2023 (pré-férias) e março de 2024 (pós-férias). Nessa escola, cerca de 72% dos alunos têm ambos os pais com diploma universitário e 91% possuem pelo menos um dos pais com formação superior.

Participaram do estudo escolares do 2° ao 5° ano do Ensino Fundamental I. Na coleta realizada em dezembro de 2022 os escolares se encontravam do 2° ao 5° ano, já em 2023 os mesmos se encontravam do 3° ao 6° ano e em 2024 do 4º ao 7º ano. Essa linha de progressão pode ser visualizada na Figura 1.

**Figura 1:** Linha de progressão de coleta de dados em quatro períodos de tempo, com duas férias

Os critérios de elegibilidade considerados para a amostra deste estudo foram: estar matriculado na mesma escola participante durante o período de coleta da pesquisa, assinatura pelos pais do Termo de Consentimento Livre e Esclarecido (TCLE) e pelos participantes do Termo de Assentimento Livre e Esclarecido (TALE). Como critério de exclusão foi considerada apenas a não participação em alguma avaliação (pré ou pós férias). A seleção dos alunos foi feita a partir do desejo dos alunos e dos pais em participar da avaliação.

### Caracterização da amostra

Foram incluídos neste estudo 98 estudantes do segundo ao quinto ano do Ensino Fundamental I que realizaram avaliação da fluência leitora em dezembro de 2022, março e dezembro de 2023 e março de 2024. Desses 98 estudantes, 31 eram do 2º ano, 23 do 3º ano, 15 do 4º ano e 24 do 5º ano.

### Procedimentos

As gravações das leituras foram obtidas presencialmente, na própria escola, de forma individual, na biblioteca e/ou em salas mais afastadas de fontes de ruídos. Utilizou-se um laptop conectado a um microfone unidirecional e o *software Praat* para gravação e síntese dos áudios que continham a leitura oral (em voz alta) feita pelos alunos. Antes das leituras, os participantes receberam a instrução de ler o texto em voz alta, começando pelo título, e quaisquer dúvidas sobre o texto ou como realizar a leitura foram esclarecidos; em seguida, os alunos executaram a leitura do texto.

Para avaliar a leitura oral, foram utilizados textos do material “Avaliação da Compreensão Leitora de Textos Expositivos”^(20)^, composto por dois textos padronizados e apropriados para cada ano escolar. Os textos apresentados aos alunos foram selecionados respeitando o ano escolar de cada grupo, com a escolha do texto com menor quantidade de palavras para o começo do ano e o texto um pouco maior para a avaliação do final do ano. Assim, em 2022, a turma do 2º ano leu um texto apropriado para seu nível. No ano seguinte (2023), já no 3º ano, os mesmos alunos leram os dois textos correspondentes a essa etapa. Em 2024, no 4º ano, a turma leu um dos textos indicados para essa série. Para evitar a influência do contato prévio com o texto, um novo texto foi aplicado em cada avaliação.

O parâmetro analisado nas gravações das leituras foi a quantidade de palavras lidas por minuto (PPM) – velocidade de leitura. As análises dos áudios foram realizadas pelo *software* Lepic^(21)^. Essa ferramenta gera um gráfico que representa o desempenho da turma, acompanhado por uma medida de desvio padrão que classifica os leitores conforme o desempenho médio da turma em excelentes (acima do desvio padrão), dentro do esperado (dentro do desvio padrão) e sob atenção (abaixo do desvio padrão). Na Figura 2 apresenta-se um exemplo do gráfico gerado pelo *software*. Os dados exibidos representam os resultados de velocidade de leitura de uma turma em um momento específico de avaliação, de forma a demonstrar a funcionalidade do programa. Os resultados são obtidos em palavras por minutos e foram armazenados em planilha Excel.

**Figura 2:** Exemplo gráfico de desempenho por turma, com as medidas de velocidade de leitura, gerado pelo *software* Lepic . Os nomes dos escolares estão protegidos sob a barra branca

Realizou-se a análise descritiva dos dados por meio de medidas de tendência central (média) e variabilidade (desvio padrão) para a variável contínua (velocidade de leitura - PPM). A distribuição quanto à normalidade das variáveis contínuas foi avaliada pelo teste Shapiro-Wilk, o qual indicou distribuição normal para a variável estudada.

Foi realizada a análise do seguimento do desempenho de fluência leitora dos alunos do 2° ano ao 5° ano do Ensino Fundamental. Comparou-se o desempenho dos mesmos alunos em dezembro/2022 e março/2023, março/2023 e dezembro/2023 e dezembro/2023 e março/2024 e a progressão da velocidade de leitura entre os anos escolares. Na coleta realizada em março de 2023 e 2024, respectivamente, os alunos se encontravam do 3° ao 6° ano e do 4º ao 7º ano. Utilizou-se o teste T pareado para a avaliação do desempenho de leitura nos diferentes momentos e o teste T para a análise da progressão. Considerou-se o nível de significância de 0,05. Para calcular o tamanho de efeito, decidiu-se agrupar os anos escolares e foi calculado a diferença entre os períodos em termos de desvio padrão por meio do teste d de Cohen, com a seguinte interpretação^(22)^:

- 0,00 - 0,19: Muito pequeno;
- 0,2 - 0,49: Pequeno;
- 0,5 - 0,79: Médio;
- A partir de 0,80: Grande.

## RESULTADOS

### Desempenho de leitura

As Tabelas 1, 2 e 3 apresentam o desempenho dos alunos quanto à velocidade de leitura nos períodos avaliados. Nas Tabelas 1 e 2 os resultados evidenciam que houve diferença com relevância estatística ao comparar o desempenho dos alunos nos períodos pré e pós-férias, indicando declínio do desempenho entre dezembro de 2022 (pré-férias) e março de 2023 (pós-férias) e entre dezembro de 2023 (pré-férias) e março de 2024 (pós-férias).

**Tabela 1:** Desempenho dos alunos quanto ao PPM em dezembro 2022 e março de 2023

Ano escolar	N	Período de avaliação	Média	Desvio padrão	PPM		
					Estatística	Graus de liberdade	Valor de p*
**2° ano**	31	**dez/22**	88,9	25,5	-2,11	30,0	0,043
		**mar/23**	94,2	26,8			
**3° ano**	23	**dez/22**	112,4	30,5	7,14	22,0	<0,001
		**mar/23**	88,4	23,1			
**4° ano**	15	**dez/22**	136,3	17,5	6,14	14,0	<0,001
		**mar/23**	112,1	19,0			
**5° ano**	29	**dez/22**	140,3	22,3	10,39	28,0	<0,001
		**mar/23**	113,5	20,1			

Legenda: N = número de estudantes

* Teste T pareado

**Tabela 2:** Desempenho dos alunos quanto ao PPM em dezembro 2023 e março de 2024

Ano escolar	N	Período de avaliação	Média	Desvio padrão	PPM		
					Estatística	Graus de liberdade	Valor de p*
**4° ano**	31	**dez/23**	108,6	20,6	12,22	30,0	<0,001
		**mar/24**	88,8	21,6			
**5° ano**	23	**dez/23**	116,2	26,1	4,81	22,0	<0,001
		**mar/24**	103,6	22,5			
**6° ano**	15	**dez/23**	140,5	19,6	6,63	14,0	<0,001
		**mar/24**	124,0	18,0			
**7° ano**	29	**dez/23**	134,5	20,6	3,46	28,0	0,002
		**mar/24**	128,2	19,1			

Legenda: N = número de estudantes

* Teste T pareado

**Tabela 3:** Desempenho dos alunos quanto ao PPM em março e dezembro de 2023

Ano escolar	N	Período de avaliação	Média	Desvio padrão	PPM		
					Estatística	Graus de liberdade	Valor de p*
**3° ano**	31	**mar/23**	94,2	26,8	-5,60	30,0	<0,001
		**dez/23**	108,6	20,6			
**4° ano**	23	**mar/23**	88,4	23,1	-10,64	22,0	<0,001
		**dez/23**	116,2	26,1			
**5° ano**	15	**mar/23**	112,1	19,0	-10,13	14,0	<0,001
		**dez/23**	140,5	19,6			
**6° ano**	29	**mar/23**	113,5	20,1	-9,21	28,0	<0,001
		**dez/23**	134,5	20,6			

Legenda: N = número de estudantes

* Teste T pareado

Já na Tabela 3, a análise compara os resultados de março e dezembro de 2023 e evidencia uma melhora no desempenho dos escolares entre o início e o fim do ano escolar.

Essa perda da aprendizagem durante as férias pode ser visualizada na Figura 3, em que foram subtraídos os valores de março de 2023 (pós-férias) pelos de dezembro de 2022 (pré-férias) (2º ao 5º ano) e os de março de 2024 (pós-férias) pelos de dezembro de 2023 (pré-férias) (3º ao 6º ano).

**Figura 3:** Diferença entre o desempenho da fluência leitora entre os períodos pós-férias (março de 2023 e março de 2024) e pré-férias (dezembro de 2022 e dezembro de 2023) em cada ano escolar

### Progressão da fluência de leitura

Na Figura 4 é possível observar a progressão da habilidade leitora de acordo com o ano escolar em 2022, 2023 e 2024. Em 2022, os resultados evidenciam diferença com estatística significativa nas comparações entre o 2º e o 3º ano e entre o 3º e o 4º ano. Quanto aos resultados de 2023, no qual os alunos do ano anterior estavam entre o 3º e o 6º ano, é evidenciado resultado com diferença estatística apenas na comparação entre o 4º e o 5º ano. Em 2024, os resultados indicam diferença estatística significativa entre o 4º e o 5º ano e entre o 5º e o 6º ano.

**Figura 4:** Comparação da velocidade de leitura entre os anos escolares em, respectivamente, dezembro de 2022, dezembro de 2023 e março de 2024

Legenda: *valores de p com estatística significativa (Teste T de Student); valor de p < 0,05

### Tamanho do efeito

Conforme Tabela 4, o tamanho do efeito nas diferenças entre grupos mostrou maior peso na comparação entre março e dezembro de 2023.

**Tabela 4:** Valores dos resultados do tamanho de feito por meio do teste d de Cohen

Períodos de avaliação	PPM - Dez. 2022	PPM - Mar. 2023	PPM - Dez. 2023	PPM - Mar. 2024
**PPM - Dez. 2022**	-	0,52	0,2	0,2
**PPM - Mar. 2023**	0,52	-	0,88	0,3
**PPM - Dez. 2023**	0,2	0,88	-	0,3
**PPM - Mar. 2024**	0,2	0,3	0,3	-

## DISCUSSÃO

O objetivo deste estudo foi avaliar o Efeito das Férias sobre a leitura^(5,6,19)^, no processo de desenvolvimento da fluência leitora dos escolares do Ensino Fundamental I, de uma escola particular situada em Belo Horizonte, Minas Gerais.

Embora as férias tenham a capacidade de oferecer oportunidades para estimular o desenvolvimento e o fortalecimento da fluência leitora, alguns alunos não são expostos à essa estimulação. Esse tempo de inatividade pode levar a diminuição temporária na prática de leitura, o que pode afetar negativamente a fluência leitora^(3,4)^.

Nesta pesquisa, observou-se queda na velocidade de leitura ao comparar os momentos pré e pós férias. Conforme apresentado na Tabela 1 e na Tabela 2, é possível verificar que a velocidade de leitura do 2° ano se manteve estável. Entretanto, nas turmas do 3°, 4° e 5° ano, houve uma piora significativa (p < 0,05), o que se supõe estar associado ao Efeito das Férias^(5)^, resultado também observado por Alves et al.^(7)^ e por Menard e Wilson^(3)^, que descrevem, em seu estudo, a redução na velocidade de leitura após as férias, associada tanto à ausência de prática quanto à menor exposição a textos.

Por outra perspectiva, a meta 5 do Plano Nacional de Alfabetização (PNE)^(23)^, prevê que todas as crianças, no máximo, até o final do 3º ano do Ensino Fundamental sejam alfabetizadas. Sendo assim, o 2° ano, por ter sido alfabetizado recentemente, pode ter recebido um maior estímulo dos pais para praticar a leitura nas férias. Dessa forma, o fato dessa turma não sofrer queda de desempenho no pós-férias pode ser explicado pelo maior incentivo e exposição à leitura durante esse período. Além disso, esses alunos foram alfabetizados no 2° período da Educação Infantil ou 1° ano do Ensino Fundamental I durante a pandemia da Covid-19, em aulas remotas, o que ocasionou um maior monitoramento e atenção da família e da escola para manter o nível de leitura. Justifica-se, assim, a manutenção da fluência leitora dessa população.

A Figura 3 apresentou, respectivamente, a diferença entre as médias de dezembro de 2022 e março de 2023 e dezembro de 2023 e março de 2024. Observa-se que o 2° ano, que inicialmente apresentava menor impacto em decorrência do efeito das férias, passou a exibir um impacto mais acentuado no ano subsequente. Tal fenômeno pode indicar uma variação na continuidade ou na intensidade das atividades educacionais entre os períodos de férias. Isto é, se o aluno deixou de receber algum apoio ou estímulo familiar entre dezembro de 2023 e março de 2024, isso pode ter contribuído para atenuar os efeitos negativos associados às férias, levando a piora no desempenho da fluência leitora^(1,7)^.

Apesar da queda no desempenho de leitura dos alunos nos períodos de férias, observa-se, na Tabela 3, que esses mesmos alunos apresentaram evolução da habilidade ao longo do ano. Comparando os resultados entre março e dezembro de 2023, todos os anos escolares obtiveram resultados superiores, com diferença estatística significativa, na segunda avaliação, o que já é esperado de acordo com a literatura^(11,24)^.

Em relação à progressão dos anos escolares, as Tabelas 1, 2 e 3 evidenciam que, com a passagem dos anos escolares há maior média de palavras lidas por minuto (PPM), isto é, há evolução da velocidade de leitura^(11)^. Essa evolução é reforçada pela Figura 4. No primeiro gráfico, com os resultados de 2022, há diferença com estatística significativa entre as turmas do 2º e 3º ano (p = 0,003) e do 3º e 4º ano (p = 0,010). Em 2023, há diferença com estatística significativa entre o 4º e o 5º ano (p = 0,004). Já em 2024, há resultados com diferença estatística entre as turmas de 4º e 5º ano (p = 0,018) e 5º e 6º ano (p = 0,005). Por fim, observa-se que os últimos anos não apresentaram resultados com diferença estatística em nenhuma das análises.

Quanto à análise do tamanho do efeito, apresentado na Tabela 4, os resultados indicaram que o efeito foi maior na comparação entre março e dezembro de 2023 (d=0,88), o que sugere uma diferença substancial na fluência de leitura ao longo do ano letivo. Esse achado pode refletir o impacto do período escolar na consolidação das habilidades de leitura, com avanços significativos entre o início e o final do ano.

Por outro lado, os valores mais baixos observados nas comparações envolvendo os meses de março (variando entre 0,2 e 0,3) indicam um efeito reduzido ou mesmo uma possível estagnação no desempenho leitor após o período de férias. Esse padrão pode estar relacionado ao conhecido declínio de aprendizagem durante o período de férias (*Summer Learning Loss*), fenômeno em que habilidades adquiridas durante o ano letivo tendem a sofrer regressão na ausência de prática regular. Esses achados corroboram os resultados mencionados anteriormente neste estudo e reforçam a importância de estratégias pedagógicas que incentivem a manutenção da leitura durante as férias, visando mitigar perdas e favorecer uma retomada mais eficiente das habilidades no início do ano letivo.

A discrepância apresentada entre os anos escolares ressalta a necessidade de pesquisas adicionais para entender melhor os fatores que contribuem para a estabilidade ou piora das habilidades de leitura durante as férias em diferentes estágios de desenvolvimento. Compreender esses fatores pode ajudar os educadores a desenvolverem estratégias mais eficazes para minimizar o impacto das férias na aprendizagem dos alunos.

O declínio das habilidades de leitura durante as férias também é influenciado pelo contexto socioeconômico. Estudos^(18,19)^ demonstram que o Efeito das Férias é significativamente maior em crianças pertencentes à classe de renda baixa, o que contribui para o aumento da disparidade de desempenho na volta às aulas^(4)^. As crianças de famílias com recursos financeiros limitados podem ter acesso reduzido a materiais de leitura, como livros e revistas. Além disso, essas famílias podem ter menos tempo e recursos para se dedicar a atividades educacionais durante as férias. Como resultado, as desigualdades no acesso e na participação em atividades de leitura durante as férias podem contribuir para aumentar as disparidades de aprendizado entre os alunos de diferentes origens socioeconômicas.

A relação entre fluência leitora e condição socioeconômica não foi levada em consideração neste estudo, por ter sido realizado em apenas uma escola de rede privada. Trata-se, portanto, de uma limitação. No entanto, essa restrição também permite uma análise mais específica do Efeito das Férias na fluência de leitura, já que o número reduzido de variáveis facilita a observação desse fenômeno. Além disso, o hábito de leitura entre os alunos da amostra não foi investigado, caracterizando-se como outra limitação do estudo.

A comparação entre turmas de diferentes séries, como o 2º e o 3º ano, pode não ser completamente representativa, pois a diferença de apenas três meses pode não ser suficiente para que os alunos do 2º ano atinjam a competência de leitura esperada para o 3º ano. Isso pode ter influenciado as medições e, consequentemente, os resultados obtidos.

Sugere-se que sejam conduzidas pesquisas mais robustas, com amostras mais representativas de diferentes tipos de escolas e outras regiões do país, para que haja generalização dos resultados do efeito das férias no Brasil. Da mesma forma, a investigação dos hábitos de leitura dos participantes e seus familiares deverá ser conduzida em pesquisas futuras.

A discussão sobre a diferença na estabilidade da velocidade de leitura entre as séries destaca a complexidade do fenômeno do Efeito das Férias e ressalta a importância de abordagens diferenciadas para apoiar o desenvolvimento contínuo das habilidades de leitura dos alunos em todas as idades e níveis de ensino.

Os resultados nos levam a refletir sobre a importância de intervenções educacionais direcionadas para remediar a regressão da leitura durante as férias. Além disso, é de suma importância a orientação familiar quanto ao hábito de leitura. Estratégias como programas de leitura de verão, acesso a recursos educacionais online e apoio individualizado podem ser cruciais para manter e fortalecer as habilidades de leitura dos alunos durante os períodos de pausa na instrução formal, desde que sejam bem planejados, tenham boa adesão e forneçam tempo suficiente para o aprendizado^(2,19,25-27)^. Desse modo, a divulgação dos resultados desta pesquisa pode inspirar ações capazes de desenvolver esta importante prática - a leitura, sendo estimulada também no período das férias escolares, de forma prazerosa.

## CONCLUSÃO

O presente estudo evidenciou o impacto negativo do Efeito das Férias no desempenho da fluência leitora de escolares do Ensino Fundamental I, que apresentaram um declínio na habilidade após o período das férias escolares prolongadas.

Os resultados da turma do 2º ano reforçam esse achado, visto que, no primeiro momento, a turma manteve as medidas de fluência leitora estáveis, logo após a alfabetização. No entanto, foi observada queda no desempenho desses alunos nos anos subsequentes, o que pode ser atribuído à redução do estímulo e à menor atenção dedicada a esses estudantes durante as férias.
